# Knowledge, Attitude, and Practice Toward Diabetes Mellitus and Their Association With Socioeconomic Status Among Patients With Type 2 Diabetes Mellitus in Saudi Arabia

**DOI:** 10.7759/cureus.39641

**Published:** 2023-05-29

**Authors:** Abdulaziz Y Almousa, Osamah A Hakami, Rayan A Qutob, Abdullah H Alghamdi, Abdullah A Alaryni, Yousef M Alammari, Khalid M Al Harbi, Meshal A Alyousef, Mohammad F Amlih, ‏Mohammad A ‏Althnayan, ‏Mohannad B ‏Almutairy

**Affiliations:** 1 College of Medicine, Imam Mohammad Ibn Saud Islamic University, Riyadh, SAU; 2 Department of Internal Medicine, Imam Mohammad Ibn Saud Islamic University, Riyadh, SAU; 3 College of Medicine, ‏Imam Mohammad Ibn Saud Islamic University, Riyadh, SAU

**Keywords:** socioeconomic status, association, practices, behavior, knowledge, type 2 diabetes mellitus

## Abstract

Objectives: The objective is to assess knowledge, attitude, and practice toward diabetes mellitus (T2DM) and its association with socioeconomic status among adult patients with T2DM.

Methods: This cross-sectional study used the validated “Diabetes Knowledge Test (DKT)” questionnaire obtained from the Michigan Diabetes Research Center. A translated copy into Arabic has been validated and used in another study. The questionnaire was created on Google Forms and distributed through digital platforms to collect data from patients with T2DM in Saudi Arabia.

Results: In this study, the majority were female (63.4%), and Saudi Arabians (96.5%), among them 23.7% lived in Riyadh, and 42.8% were from the central region. As for marital status, 60.5% were married, 28.4% were single, and 11.1% were divorced or widowed. 58.9% had college/higher degrees, and 45.8% were unemployed. Furthermore, the majority (47.1%) reported having a salary of less than 5,000 Saudi Riyals per month. 55.1% of participants lived in villas, while 46.6% had 6-10 people living in their household. Generalized linear model (GLM) findings showed that age, marital status, level of education, monthly income, and accommodation are significantly correlated with the level of knowledge.

Conclusion: Findings indicated a high level of knowledge, positive behavior, and good adherence to practice among patients with T2DM. GLM findings showed that age, marital status, level of education, monthly income, and accommodation are significantly correlated with the level of knowledge. Researchers suggest that effective health education interventions are needed to improve diabetes knowledge, behavior, and practices, particularly regarding lifestyle modifications and dietary management.

## Introduction

Diabetes mellitus (DM) is a long-term disorder characterized by abnormally high blood glucose levels. The most common forms of diabetes are type 2 DM (T2DM) and type 1 DM (T1DM). T2DM is characterized by hyperglycemia, insulin resistance, and relative insulin secretion impairment. Furthermore, it is the most prevalent form of diabetes, which poses a threat to global public health. T2DM pathogenesis is heterogeneous, with insulin resistance being influenced by several components. Key contributing factors include genetic influence and environmental aspects (e.g., obesity, sedentary lifestyle) [1].

Diabetes management is a long-term process; therefore, patient motivation is essential. Patients must have a basic understanding of diabetes, which can contribute to a more positive attitude and practice in the long run. This, in turn, can facilitate the early detection of disease and the reduction of complications [2].

In a 2019 study by Saeedi [3], DM is estimated to affect 9.3% (463 million people) of the global population, with projections indicating an increase to 10.2% (578 million) by 2030 and 10.9% (700 million) by 2045. The study revealed a higher prevalence of diabetes in urban areas (10.8%) compared to rural regions (7.2%), as well as in high-income countries (10.4%) compared to low-income ones (4.0%). Notably, Saudi Arabia ranks seventh globally and second in the Middle East in terms of diabetes prevalence [4]. Another study reported an estimated three million patients with prediabetes and seven million with DM in Saudi Arabia [5].

Education is one of the primary interventions employed by healthcare workers to improve patients' knowledge. Additionally, interventions such as increasing motivation and promoting self-management are crucial in assisting patients with diabetes to maintain optimal glycemic control. Studies have demonstrated that patients who possess knowledge about diabetes self-care achieve better long-term glycemic control [6]. Understanding glycemic control helps individuals comprehend the risks associated with diabetes and motivates them to seek appropriate care and treatment, thus enabling effective disease management [7].

The objective of this cross-sectional study is to objectively assess the knowledge, attitude, and practice of adult patients with T2DM regarding diabetes, while also exploring the association with socioeconomic status. The study aims to identify areas where knowledge is lacking and develop future online educational activities to target those areas.

## Materials and methods

This cross-sectional study utilized an online questionnaire adapted from a previous study by Abdulrahman et al., who employed the "Diabetes Knowledge Test (DKT)" questionnaire obtained from the Michigan Diabetes Research Center and translated into Arabic. The corresponding author granted permission to use it in our project [8]. The demographic section was modified to align with our study population. The questionnaire was created on Google Forms® and divided into three sections: demographics, knowledge, and attitude and practice. It was then distributed to the participants via digital platforms by data collectors to collect the data between July 2022 and November 2022, employing a convenience sampling technique. Informed consent and declaration of the authenticity of the information were included at the beginning of the survey, and participation was voluntary. A minimum sample size of 934 was determined based on a 95% confidence level, a margin of error of 2.7%, and a population proportion of 23%. The study participants consisted of Saudi and non-Saudi adults (>18 years) diagnosed with T2DM, residing in Saudi Arabia during the study period, and receiving healthcare from either public or private healthcare facilities, regardless of their disease duration. Exclusion criteria included T1DM, pregnant women, and participants residing outside Saudi Arabia. Data were analyzed using SPSS (IBM Corp., Armonk, NY), and the results were reviewed, tabulated, and compared for statistical significance. A p-value of <=0.05 was considered statistically significant. The dependent variables are patients' knowledge, attitude, and practice, while the independent variables are socio-demographic characteristics.

Ethics approval and consent to participate

The ethical standards formed by the institutional and national research committees, the 1964 Helsinki Declaration and its associated regulations, or comparable ethical principles were followed in this cross-sectional study that involved human subjects. The Institutional Review Board at Imam Mohammad ibn Saud Islamic University approved this study (IRB approval No. 298/2022). All study participants provided written consent before agreeing to participate.

## Results

In this study, 936 patients with T2DM in Saudi Arabia participated in the online survey. Table 1 presents the characteristics of the study sample, with the majority being female (63.4%), Saudi Arabians (96.5%), residing in Riyadh (23.7%), and from the Central region (42.8%). In terms of marital status, 60.5% were married, 28.4% were single, and 11.1% were divorced or widowed. Regarding educational level and occupation, 58.9% had a college degree or higher, and 45.8% were unemployed. When asked about their monthly income, the majority (47.9%) reported having less than 5,000 Saudi Riyals per month. Additionally, 55.1% of participants lived in villas, while 46.6% had six to 10 residents in their households.

**Table 1:** Socio-demographic characteristics of the 936 study participants.

Demographics		Count	%
Total		936	100.0
Gender	Male	343	36.6
	Female	593	63.4
Age	18-25	229	24.5
	26-35	134	14.3
	36-45	172	18.4
	46-55	198	21.2
	56-65	132	14.1
	More than 65	71	7.6
Nationality	Saudi	903	96.5
	Non-Saudi	33	3.5
City of residence	Riyadh	222	23.7
	Makkah	195	20.8
	Madinah	41	4.4
	Eastern Province	79	8.4
	Qassim	179	19.1
	Hail	28	3.0
	Tabuk	3	0.3
	Al-Jawf	1	0.1
	Northern Borders	5	0.5
	Al-Baha	5	0.5
	Asir	148	15.8
	Jazan	8	0.9
	Najran	22	2.4
Region of residence	Northern region	37	4.0
	Western region	241	25.7
	Central region	401	42.8
	Eastern region	79	8.4
	Southern region	178	19.0
Marital status	Single	266	28.4
	Married	566	60.5
	Divorced/Widowed	104	11.1
Educational level	Primary school.	169	18.1
	High/Secondary school	216	23.1
	College/higher degrees	551	58.9
Occupation	Unemployed	429	45.8
	Government employee	279	29.8
	Private/freelance	89	9.5
	Retired	139	14.9
Monthly Income (Saudi Riyals)	Less than 5,000	448	47.9
	5,000-10,000	216	23.1
	11,000-15,000	144	15.4
	More than 15,000	128	13.7
Accommodation type	Apartment for rent	159	17.0
	Own apartment	184	19.7
	Villa/house for rent	77	8.2
	Villa/owner's house	516	55.1
The number of residents	1-2	89	9.5
	3-5	332	35.5
	6-10	436	46.6
	More than 10	79	8.4

Table 2 demonstrates information on the duration of illness, family history, medical care, and the settings in which participants monitored their diabetes. It was found that 20.3% of the patients had been diagnosed with diabetes for one to five years, while 7.9% had a duration of 16 to 20 years. A significant proportion of participants (74.7%) had a family history of diabetes. Furthermore, 42.3% received medical care at an endocrinology or diabetes clinic, while 36.6% were monitored at a family medicine clinic or general practitioner. As for the settings where they monitored their diabetes, 41.9% reported hospitals or diabetes centers affiliated with the ministry of health.

**Table 2:** Medical history of patients with type 2 diabetes.

		Count	%
		936	100.0
Duration of diabetes? (in years)	Undiagnosed	29	3.1
	<1 year	275	29.4
	1-5 year	190	20.3
	6-10 year	149	15.9
	11-15 year	101	10.8
	16-20 year	74	7.9
	More than 20 years	118	12.6
Family History of Diabetes:	Yes	699	74.7
	No	237	25.3
Who provides medical care for diabetes?	Endocrinology or diabetes clinic	396	42.3
	Family medicine clinic or general practitioner	343	36.6
	No one	197	21.0
In which medical set-up do you mainly monitor your diabetes-related health condition?	A hospital or diabetes center affiliated with ministry of health.	392	41.9
	A primary care center affiliated with ministry of health.	186	19.9
	Medical city.	48	5.1
	Private clinic.	62	6.6
	Clinic in a private hospital.	98	10.5
	I do not follow any clinic.	150	16.0

Table 3 shows the level of knowledge regarding T2DM indicated by the percentage of correct answers. The majority of participants demonstrated accurate knowledge regarding various aspects of diabetes. Specifically, 51.2% had correct knowledge about diet in diabetes, 55.0% identified foods high in carbohydrates correctly, 60.4% understood the glycosylated hemoglobin (HbA1c) test, and 72.5% were knowledgeable about home glucose testing. Additionally, a significant number of participants exhibited correct understanding of factors influencing blood glucose levels, such as exercise (80.0%), infection (61.2%), and consuming low-fat foods (75.2%). When it comes to identifying numbness and tingling as a symptom of neuropathy, 79.0% responded accurately. Furthermore, 64.6% correctly identified variables associated with diabetes, and 81.0% recognized low blood glucose reactions resulting from taking rapid-acting insulin. Regarding practices, 66.9% knew the appropriate foot care, 51.7% were aware of what to do when insulin was not taken during breakfast, and 62.5% knew the appropriate actions to take during a low glucose reaction. Many participants also correctly answered questions about the causes of low blood glucose (61.2%), the expected blood glucose level in individuals who took insulin but skipped breakfast (73.4%), and the factors leading to high blood glucose (62.2%).

**Table 3:** Level of knowledge regarding type 2 diabetes (% of correct answers), n=936

Question	Count	%
The diabetes diet is	479	51.2
Which of the following is highest in carbohydrate?	515	55.0
Which of the following is highest in fat?	372	39.7
Which of the following is a “free food”?	304	32.5
A1C is a measure of your average blood glucose level for the past	565	60.4
Which is the best method for home glucose testing?	679	72.5
What effect does unsweetened fruit juice have on blood glucose?	453	48.4
Which should not be used to treat a low blood glucose?	304	32.5
For a person in good control, what effect does exercise have on blood glucose?	749	80.0
What effect will an infection most likely have on blood glucose?	573	61.2
The best way to take care of your feet is to	626	66.9
Eating foods lower in fat decreases your risk for	704	75.2
Numbness and tingling may be symptoms of	739	79.0
Which of the following is usually not associated with diabetes:	605	64.6
If you are sick with the flu, you should:	463	49.5
If you have taken rapid-acting insulin, you are most likely to have a low blood glucose reaction in	758	81.0
You realize just before lunch that you forgot to take your insulin at breakfast. What should you do now?	484	51.7
If you are beginning to have a low blood glucose reaction, you should:	585	62.5
A low blood glucose reaction may be caused by	573	61.2
If you take your morning insulin but skip breakfast, your blood glucose level will usually:	687	73.4
High blood glucose may be caused by	582	62.2
A low blood glucose reaction may be caused by	451	48.2

The participants' level of knowledge regarding type 2 diabetes was assessed using a scoring system of 0 and 1, representing incorrect and correct answers, respectively. Table 4 illustrates that the knowledge level among participants had a mean score of 59.59 ± 19. Additionally, the majority of patients (n=91, 9.7%) achieved a score of 77.7, while only two (0.2%) patients scored 100.

**Table 4:** Overall knowledge scores of the 936 patients with T2DM Total score of 100: Minimum: 9.09, Maximum: 100, mean: 59 + SD: 19.8

Score	N	%
100	936	100.0
9.09	3	0.3
13.64	8	0.9
18.18	12	1.3
22.73	27	2.9
27.27	30	3.2
31.82	47	5.0
36.36	42	4.5
40.91	57	6.1
45.45	44	4.7
50.00	49	5.2
54.55	69	7.4
59.09	74	7.9
63.64	60	6.4
68.18	86	9.2
72.73	86	9.2
77.27	91	9.7
81.82	67	7.2
86.36	47	5.0
90.91	21	2.2
95.45	14	1.5
100.00	2	0.2

Table 5 provides an overview of the behaviors and practices of the 936 patients in the study. The highest-rated behavior was the commitment to taking prescribed medication according to the doctor's instructions without negligence, with a mean score of 3.97 ± 1.3. Following that, the statement “I hope that the medical staff will provide free educational courses and seminars (physicians and health educators)” (3.84 ± 1.4) and “I monitor my blood sugar using a home device as per my doctor's advice” (3.82 ± 1.4) ranked as the second and third highest, respectively. The three lowest-rated behaviors, in increasing order, were “I had traveled before and forgot to take my medication with me” (2.50 ± 1.6), “During the past two weeks, there have been days when I forgot to take some diabetes medication (2.61 ± 1.6),” and “You have previously stopped using certain medicines without telling your doctor because of their side effects” (2.65 ± 1.6).

**Table 5:** Behavior and practice of the diabetic patient

	Min	Max	Mean	SD
I receive sufficient information about diabetes from the medical staff; Physician and health education.	1	5	3.78	1.4
I rely on the advice of friends and colleagues regarding my diabetes.	1	5	3.07	1.5
I rely on the information and advice I receive through social media (WhatsApp, Twitter, Snapchat, etc.) regarding diabetes.	1	5	2.91	1.5
Social media information and advice is a primary source for me, due to the poor health education of the medical staff and the crowded hospitals.	1	5	2.95	1.5
I tried and am still trying to lose weight.	1	5	3.60	1.4
I visit the doctor every 3-6 months for regular check-ups.	1	5	3.79	1.4
I visit the ophthalmologist at least once a year to examine the retina and the fundus of the eye.	1	5	3.44	1.5
I check my feet regularly and see a foot specialist if necessary.	1	5	3.29	1.5
I monitor my blood sugar using a home device as per my doctor's advice.	1	5	3.82	1.4
At every visit, the doctor asks me about my diet and exercise.	1	5	3.76	1.4
I do at least a brisk walk every day for half an hour	1	5	3.40	1.5
I follow a diabetes Appropriate diet under the supervision of a nutritionist.	1	5	3.18	1.5
I receive the necessary vaccinations on a regular basis, such as the seasonal flu vaccination every year.	1	5	3.28	1.6
I commit to taking the medication prescribed to me according to the doctor’s instructions, without negligence.	1	5	3.97	1.3
I stop taking some medications if I feel my blood sugar level is under control.	1	5	2.91	1.6
I frequently have difficulty remembering to take my diabetes medications.	1	5	2.73	1.5
During the past two weeks, there have been days when I forgot to take some diabetes medication.	1	5	2.61	1.5
You have previously stopped using certain medicines without telling your doctor because of their side effects.	1	5	2.65	1.6
I have travelled before and forgot to take my medication with me.	1	5	2.50	1.6
I hope that free educational courses and seminars will be provided by the medical staff. Physician and health education.	1	5	3.84	1.4

Statistical correlations were conducted in this study. Table 6 presents the three highest and lowest behavior/practice points and their associations with the level of knowledge regarding T2DM. The analysis revealed that the three highest behavior/practices were positively correlated with the level of knowledge (all p < 0.001), while the three lowest behaviors showed a significant negative correlation (all p < 0.001).

**Table 6:** Association of behavior/practices with level of knowledge regarding diabetes

Correlations		Level of knowledge
Top three highest behavior/practice		
I commit to taking the medication prescribed to me according to the doctor’s instructions, without negligence.	r	0.215**
	P-value	<0.001
	N	936
I hope that free educational courses and seminars will be provided by the medical staff. Physician and health education.	r	0.143**
	P-value	<0.001
	N	936
I monitor my blood sugar using a home device as per my doctor's advice.	r	0.163**
	P-value	<0.001
	N	936
Top three lowest behavior/practice		
You have previously stopped using certain medicines without telling your doctor because of their side effects.	r	-0.245**
	P-value	<0.001
	N	936
During the past two weeks, there have been days when I forgot to take some diabetes medication.	r	-0.210**
	P-value	<0.001
	N	936
I have travelled before and forgot to take my medication with me.	r	-0.300**
	P-value	<0.001
	N	936
**Correlation is significant at the 0.01 level (2-tailed).		

The participants' socio-demographic characteristics, as shown in Table 7, were examined for their correlation with the level of knowledge. It was found that gender exhibited a significant correlation with the knowledge level using Welch's t-test at a significance level of 0.05. Age (p < 0.001), marital status (p < 0.001), and occupation (p < 0.001) were also significantly associated with the level of knowledge according to One-Way ANOVA at a significance level of <0.05, with Games-Howell used as the post-hoc test. Furthermore, housing area (p = 0.021), educational level (p = 0.004), monthly income (p < 0.001), accommodation type (p < 0.001), and the number of residents (p = 0.008) exhibited a significant correlation with the level of knowledge using One-Way ANOVA at a significance level of <0.05, with LSD used as the post-hoc test.

**Table 7:** Association of socio-demographic characteristics with level of knowledge regarding diabetes a-significant using Welch's t-test at 0.05 level. b-significant using One-Way ANOVA at <0.05 level. c-Post-hoc Test = LSD. d-Post-hoc Test = Games-Howell. *CAPITAL letters indicates Post-Hoc multiple pairing summary indicator. Having the same letter means the same measure statistically.

Demographics		Total	Level of knowledge	P-value
Gender	Male	343	57.06 ± 21.7	0.006^a^
	Female	593	60.89 ± 18.5	
Age	18-25	229	50.99 ± 21.9^AB^	<0.001^b,d^
	26-35	134	55.77 ± 18.9^BC^	
	36-45	172	60.02 ± 18.2^C^	
	46-55	198	64.51 ± 18.4^CD^	
	56-65	132	67.60 ± 16.5^D^	
	More than 65	71	63.57 ± 15.5^CD^	
Region of residence	Northern region	37	53.69 ± 22.4^A^	0.021^b,c^
	Western region	241	61.81 ± 18.7^B^	
	Central region	401	59.39 ± 19.6^AB^	
	Eastern region	79	62.08 ± 20.2^B^	
	Southern region	178	56.64 ± 20.5^A^	
Marital status	Single	266	51.23 ± 21.6^A^	<0.001^b,d^
	Married	566	62.82 ± 17.9^B^	
	Divorced/Widowed	104	62.50 ± 18.7^B^	
Educational level	Primary school.	169	58.80 ± 18.6^A^	0.004^b,c^
	High/Secondary school	216	55.93 ± 19.9^B^	
	College/higher degrees	551	61.10 ± 20.0^B^	
Occupation	Unemployed	429	58.51 ± 19.9^A^	<0.001^b,d^
	Government employee	279	58.98 ± 20.2^A^	
	Private/freelance	89	55.11 ± 20.0^A^	
	Retired	139	66.35 ± 17.1^B^	
Monthly Income	Less than 5,000	448	56.44 ± 19.6^A^	<0.001^b,c^
	5,000-10,000	216	59.68 ± 18.8^B^	
	11,000-15,000	144	62.97 ± 19.2^BC^	
	More than 15,000	128	65.91 ± 20.8^C^	
Accommodation type	Apartment for rent	159	55.00 ± 20.6^A^	<0.001^b,c^
	Own apartment	184	55.24 ± 19.2^A^	
	Villa/house for rent	77	55.55 ± 18.5^A^	
	Villa/owner's house	516	62.98 ± 19.3^B^	
Number of residents	1-2	89	52.81 ± 20.2^A^	0.008^b,c^
	3-5	332	59.87 ± 19.1^B^	
	6-10	436	60.63 ± 19.9^B^	
	More than 10	79	59.09 ± 20.9^B^	

The medical history of diabetes in the study population was also assessed and correlated with the level of knowledge, as presented in Table 8. It was found that family history of diabetes, duration of diabetes, the medical field where they monitored their condition, and medical care provider (all p < 0.001) were significantly associated with the level of knowledge.

**Table 8:** Associations of Medical History with Level of knowledge regarding diabetes

Diabetes mellitus (T2DM) medical history		Total	Level of knowledge	P-value
Duration of diabetes (years)?				<0.001^b,d^
	<1 year	275	52.61 ± 19.4^B^	
	1-5 year	190	59.74 ± 19.5^C^	
	6-10 year	149	61.47 ± 19.2^C^	
	11-15 year	101	65.35 ± 19.5^AC^	
	16-20 year	74	61.30 ± 21.0^AC^	
	More than 20 years	118	63.67 ± 18.0^AC^	
Family History of Diabetes:	Yes	699	60.95 ± 19.6	<0.001^a^
	No	237	55.18 ± 19.9	
Who provides medical care for diabetes?	Endocrinology or diabetes clinic	396	62.44 ± 19.7^A^	<0.001^b,c^
	Family medicine clinic or general practitioner	343	59.67 ± 19.7^A^	
	No one	197	53.23 ± 18.8^B^	
In which medical field do you mainly monitor your diabetes-related health condition?	A hospital or diabetes center affiliated with ministry of health	392	61.80 ± 18.8^A^	<0.001^b,d^
	A primary care center affiliated to a government ministry.	186	60.95 ± 17.7^A^	
	Medical city.	48	46.97 ± 22.5^B^	
	Private clinic.	62	49.49 ± 22.2^B^	
	Clinic in a private hospital.	98	67.30 ± 20.0^A^	
	I do not follow any clinic.	150	54.67 ± 18.6^B^	
^a^-significant using Independent t-test at 0.05 level. ^b^-significant using One-Way ANOVA at <0.05 level. ^c^-Post-hoc Test = LSD. ^d^-Post-hoc Test = Games-Howell. *CAPITAL letters indicates Post-Hoc multiple pairing summary indicator. Having the same letter means the same measure statistically.				

A General Linear Model (GLM) was applied at a significance level of <0.05 to analyze the responses of the study samples. The results showed that only the highest-ranked behavior (p < 0.001) and second-highest behavior (p = 0.004), as well as the highest-ranked behavior (p = 0.003) and third-lowest behavior (p < 0.001), were significantly correlated with the level of knowledge, as indicated in Table 9. Furthermore, after statistical adjustment of the data, Table 10 revealed that being in the age range of 18-25 years old (p = 0.042), belonging to the western region (p = 0.045), being single (p = 0.002), having primary education (p < 0.001) or secondary education (p = 0.002), earning a monthly income of less than 5000 SAR (p = 0.037), and residing in a rented apartment (p = 0.004), owned apartment (p < 0.001), or villa or house (p = 0.013) were significantly associated with the level of knowledge.

**Table 9:** Association of behavior and practices with level of knowledge using GLM

Dependent Variable: Level of knowledge regarding diabetes					
Parameter	B	S.E.	95% Confidence Interval		P-value
			Lower Bound	Upper Bound	
Intercept	54.525	2.521	49.577	59.473	<0.001^a^
Top 3 Highest Behavior/Practice					
I commit to taking the medication prescribed to me according to the doctor’s instructions, without negligence.	2.041	0.543	0.976	3.106	<0.001^a^
I hope that free educational courses and seminars will be provided by the medical staff. Physician and health education.	1.414	0.490	0.453	2.376	0.004^a^
I monitor my blood sugar using a home device as per my doctor's advice.	0.925	0.504	-0.064	1.915	0.067
Top 3 Lowest Behavior/Practice					
You have previously stopped using certain medicines without telling your doctor because of their side effects.	-1.471	0.491	-2.434	-0.507	0.003^a^
During the past two weeks, there have been days when I forgot to take some diabetes medication.	-0.508	0.493	-1.475	0.460	0.303
I have travelled before and forgot to take my medication with me.	-2.753	0.483	-3.701	-1.806	<0.001^a^
^a^-significant using General Linear Model(GLM) at <0.05 level.					

 

**Table 10:** Association of Socio-demographic characteristics to level of knowledge regarding diabetes mellitus using general linear model (GLM).

Dependent Variable: Level of knowledge regarding diabetes					
Parameter	B	S.E.	95% Confidence Interval		p-value
			Lower Bound	Upper Bound	
Intercept	71.363	4.073	63.369	79.357	<0.001^a^
Demographics					
Gender = Male	-1.594	1.443	-4.426	1.238	0.270
Age = 18-25	-7.068	3.471	-13.881	-0.255	0.042^a^
Age = 26-35	-4.974	3.353	-11.555	1.606	0.138
Age = 36-45	-4.015	3.126	-10.149	2.120	0.199
Age = 36-45	-0.895	2.878	-6.544	4.754	0.756
Age = 56-65	1.368	2.791	-4.111	6.846	0.624
Region of residence = Northern region	-1.757	3.356	-8.343	4.829	0.601
Region of residence = Western region	3.766	1.877	0.083	7.449	0.045^a^
Region of residence = Central region	0.539	1.681	-2.761	3.839	0.748
Region of residence = Eastern region	3.996	2.512	-0.933	8.925	0.112
Marital status = Single	-9.112	2.899	-14.802	-3.423	0.002^a^
Marital status = Married	-0.571	2.134	-4.760	3.617	0.789
Educational level = Primary school	-7.247	2.028	-11.227	-3.268	<0.001^a^
Educational level = High/Secondary school	-4.692	1.538	-7.710	-1.673	0.002^a^
Occupation = Unemployed	3.222	2.490	-1.665	8.108	0.196
Occupation = Government employee	-2.265	2.360	-6.897	2.367	0.337
Occupation = Private/freelance	-1.779	2.855	-7.383	3.825	0.533
Monthly Income = Less than 5,000	-4.993	2.389	-9.681	-0.304	0.037^a^
Monthly Income = 5,000-10,000	-2.903	2.199	-7.220	1.414	0.187
Monthly Income = 11,000-15,000	-3.290	2.281	-7.767	1.187	0.150
Accommodation type = Apartment for rent	-5.346	1.849	-8.975	-1.717	0.004^a^
Accommodation type = Own apartment	-6.947	1.699	-10.282	-3.612	<0.001^a^
Accommodation type = Villa/house for rent	-5.633	2.273	-10.093	-1.173	0.013^a^
Number of residents = 1-2	-2.200	3.020	-8.127	3.727	0.466
Number of residents = 3-5	1.953	2.425	-2.807	6.713	0.421
Number of residents = 6-10	1.998	2.291	-2.499	6.496	0.383
^a^-significant using General Linear Model (GLM) at <0.05 level.					

## Discussion

In this study, the levels of knowledge, behavior, and practices of patients with T2DM in Saudi Arabia were examined, as prior research has suggested that proper knowledge, attitudes, and behaviors are required to lower the risk of acquiring diabetes (6). Analysis of existing studies has shown that poor blood glucose control is influenced by factors such as age, education, knowledge, and duration of treatment [9]. The results of this study revealed that participants had good knowledge regarding diabetes [10,11].

A majority of the patients in this study correctly identified what a diabetes diet is 51.2% and foods that are high in carbohydrates (55.0%). This contrasts with the findings of the study by Bano et al. [12], where 81% of participants lacked knowledge about diabetic diets. Another study conducted by Sami et al. [13] in the Kingdom of Saudi Arabia showed poor dietary knowledge specifically related to carbohydrates.

The findings further revealed that most of the patients had knowledge about diabetes-related measurements or tests, such as the A1C test (60.4%) and home glucose testing (72.5%). The A1C test, also known as the hemoglobin A1C or HbA1c test, is a simple test that determines the average blood sugar levels over the past three months. It is important for diabetes diagnosis and management as it provides an understanding of glucose levels and insulin resistance [14]. Home glucose testing, on the other hand, involves blood testing to monitor glucose levels.

The majority of the participants also had sufficient knowledge regarding factors that may affect blood glucose. They correctly identified the effects of exercise (80.0%), infection (61.2%), and consuming foods lower in fat (75.2%). Studies have shown that exercise or physical activity is important for the therapeutic approach of T2D patients [15]. As it helps improve body weight, body mass index (BMI), and glucose tolerance, and reduces the risk of developing diabetes. Infections, on the other hand, can induce a stress response in the body, leading to increased levels of hormones such as cortisol and adrenaline. These hormones work against insulin, causing the body to produce more glucose, which can result in diabetes.

The current study revealed a mean knowledge score of 59.49+19, indicating a higher level of diabetes knowledge compared to studies conducted in Nepal and Fiji, where the mean (SD) knowledge scores were 11.0+3.32 and 23.3+3.25, respectively. These differences in knowledge across studies could be explained by variations in participants' socio-demographics, educational levels, and the availability of diabetes knowledge at the time. A previous study identified gender, age, and socio-economic status as risk factors for explaining discrepancies in knowledge, attitudes, and practices [6]. The majority of participants in this study had a college/higher education, which justifies their high knowledge score.

The findings revealed that patients in the current study exhibited good behavior and practices. The highest behavior was the commitment to take medication according to the doctor's instructions, with a mean score of 3.97+1.3, while the lowest behavior was stopping the use of certain medications without informing the doctor due to side effects. Statistical analysis showed that the top one highest and lowest behaviors/practices were significantly associated with the level of knowledge at a 0.01 significance level. However, after adjusting for R-squared, the results changed, and the top one highest and top three lowest behaviors/practices were significantly associated with the level of knowledge. Nevertheless, based on the mean scores ranging from 2.50 to 3.97, the participants in this study exhibited good behavior/practices. Findings from Saadia et al. [16] revealed that participants' understanding of diabetes in their study was good, but their behavior and practice were poor. Ng et al. [17] concluded in their study that variables such as sufficient knowledge and practice resulted in good disease management practices.

It was found that gender was significantly correlated with the level of knowledge using Welch's t-test at a 0.05 level. Age (p<0.001), marital status (p<0.001), and occupation (p<0.001) were also significantly associated with the level of knowledge according to one-way ANOVA at a <0.05 level, with Games-Howell as the posthoc test. Furthermore, housing area (p=0.021), educational level (p=0.004), monthly income (p<0.001), accommodation type (p<0.001), and the number of residents (p=0.008) showed a significant correlation with the level of knowledge using One-Way ANOVA at a <0.05 level, with LSD as the posthoc test.

Statistical analysis was performed in this study to determine factors associated with the level of knowledge. The GLM findings showed that age, marital status, level of education, monthly income, and accommodation were significantly correlated with the level of knowledge. Similar results were found in a study conducted in Bangladesh [18], where knowledge levels were strongly associated with education level, salary, residence, diabetic status, BMI, and attitude. In a study in Southern Benin, regression analysis showed that variables such as being female, married, educated, government/non-government employee, and having diabetes for a longer period of time were significantly related to good knowledge [1]. In a study conducted in Qatar, knowledge levels differed significantly by gender, nationality, and diabetes-related diagnosis (p<0.001), while attitude and practice levels differed significantly across all four factors (p<0.001). However, age was not found to be statistically significant [19]. In the study in Sri Lanka, gender and age were not statistically correlated with the level of knowledge [20].

This cross-sectional study has several potential limitations that should be considered. Firstly, the data on knowledge, behavior, and practices regarding type 2 diabetes were self-reported, which introduces the possibility of recall bias. Participants may have difficulty accurately recalling their knowledge or behaviors, and their responses may be influenced by social desirability, leading to overreporting of positive behaviors and underreporting of negative behaviors.

Another limitation is that the study included all type 2 diabetic patients without considering their diabetic complication history status and the level of glucose control HbA1c or self-reported home glucose testing. during the data collection period. Diabetic complications can significantly impact an individual's behavior and practices related to diabetes management [21]. Therefore, the participants' behavior and practice levels may have been influenced positively or negatively by their specific complication histories and HbA1c levels, which were not considered.

Although the study had a large number of participants, providing a representative sample of the general public with type 2 diabetes in Saudi Arabia, it is important to note that the generalizability of the findings may still be limited. The sample may not fully represent the diversity of the population, and there could be variations in knowledge, behavior, and practices among different subgroups that were not adequately captured in the study.

Lastly, since this was a cross-sectional survey, only associations between variables could be determined, and no causal relationships can be inferred. Longitudinal studies or experimental designs would be necessary to establish causal links between knowledge, behavior, and practices regarding type 2 diabetes.

Overall, while this study provides valuable insights into the knowledge, behavior, and practices of patients with type 2 diabetes in Saudi Arabia, the limitations should be taken into consideration when interpreting the results. Future research should address these limitations to further enhance our understanding of diabetes management in this population.

## Conclusions

This cross-sectional study revealed a favorable level of knowledge, behavior, and practices among participants with type 2 diabetes. The GLM analysis demonstrated significant associations between age, marital status, level of education, monthly income, and accommodation with the level of knowledge. The researchers recommend implementing effective health education interventions to enhance diabetes knowledge, behavior, and practices, particularly in relation to lifestyle modifications, dietary management, and poor socioeconomic status.
